# Characterization of feline mandibular angle fractures utilizing *in silico* model construction and fracture mapping

**DOI:** 10.3389/fvets.2025.1555190

**Published:** 2025-03-21

**Authors:** Tsung-Han Tu, Scott J. Hetzel, Jason W. Soukup

**Affiliations:** ^1^Dentistry and Oromaxillofacial Surgery, Department of Surgical Sciences, School of Veterinary Medicine, University of Wisconsin–Madison, Madison, WI, United States; ^2^Department of Biostatistics and Medical Informatics, University of Wisconsin–Madison, Madison, WI, United States

**Keywords:** feline, mandible, fracture, *in silico*, fracture mapping, mandibular angle

## Abstract

**Introduction:**

The unique and dynamic features of the feline mandibular angle make open reduction and internal fixation in this region more challenging than in the mandibular body. Visualization of fracture patterns through a fracture map can be a valuable tool for qualitative analysis of fractures in this region. In addition, fracture maps are useful in designing hardware for rigid internal fixation. The primary aim of this study was to identify possible associations between patient demographics, fracture etiologies, and fracture patterns affecting the feline mandibular angle. The secondary aim was to create fracture maps to qualitatively characterize fracture patterns.

**Methods:**

Nineteen cats with 22 mandibular angle fractures were included in this retrospective study. Medical records were reviewed and statistically analyzed. Fracture maps were created using three-dimensional in silico models derived from computed tomographic images and analyzed based on fracture categories/features (simple vs. comminuted fractures, fracture etiologies, bilateral fractures, and age).

**Results:**

No significant associations were found between dependent variables (fracture type, dorsal fracture location/fracture origin, ventral fracture location/fracture termination, mandibular foramen involvement, and displacement score) and independent variables (age, sex, and etiology). Fracture maps provided important qualitative information that was not evident from statistical analysis of patient demographics/fracture characteristics.

**Discussion:**

We conclude that in silico evaluation of fracture patterns provides important qualitative information that could not be obtain by traditional fracture characterization. In addition, the unique morphologic features of the feline mandibular angle likely play a significant role in fracture mechanics and fracture patterns.

## Introduction

Mandibular fractures account for 14.5% of all feline fractures, with caudal mandibular fractures making up 10.7% of all mandibular fractures (1). These fractures not only cause immediate pain but also can often lead to malocclusion, which can result in trauma to the soft and hard tissues of the oral cavity (2). If left untreated, mandibular fractures can lead to malunion and non-union, further contributing to malocclusion and repetitive masticatory trauma (3). The primary goal of treating oral and maxillofacial fractures is to regain normal, trauma-free masticatory function (2). Successful treatment of feline mandibular fractures has been reported using different techniques. These techniques have different indications based on the nature and location of the fracture and provide varying levels of stability (4). The possibility of iatrogenic trauma (e.g., tissue dissection), which is influenced by the age of the patient and the fracture location, should be considered with each repair technique (5). Compared to non-rigid conservative fracture stabilization methods (e.g., muzzle, modified labial button technique, and bignathic encircling and retaining device [BEARD]) and semi-rigid fracture stabilization methods (e.g., interdental wiring, intraoral splint, and interarcade fixation), open reduction and internal fixation (ORIF) provides the most stable fracture stabilization and offers a higher likelihood of achieving primary bone healing (4, 6–10). It is recommended that each fracture fragment be secured with at least two screws and engage four cortices to ensure sufficient screw purchase (11). This is generally achievable when treating mandibular fractures in human patients and large-breed dogs, as the fragments are usually large enough. This is also generally true for fractures in the caudal portion of the mandibular body in feline patients. However, the application of ORIF in more rostral fractures can be challenging due to the risk of damaging the canine tooth root.

There are several unique anatomical features of the caudal mandibular region, particularly in cats, that make this area a challenge to treat. The *mandibular angle* serves as a transition zone from a horizontal dentate mandibular body to a vertical ramus, which serves as the insertion zone for the masticatory muscles. In human anatomy, the term *mandibular angle* derives from the angular process. It is important to note that the angle is an anatomic area rather than a distinct anatomical structure. The exact definition of the mandibular angle in human literature is vague; however, the consensus is that it lies between a vertical line just distal to the mandibular third molar and a horizontal line extending from the mandibular third molar to a point ventral to the condylar process of the mandible. Any fracture of the mandible in this region, thus, can be considered a *mandibular angle fracture* (7, 12). To the authors’ knowledge, there is no veterinary literature that clearly defines the angle of the feline mandible. Even though the configuration of the feline mandible has significant differences compared to its human counterpart, they share similar basic anatomical landmarks (e.g., mandibular canal, mandibular foramina, angular process, condylar, and coronoid processes) (1, 13).

The lingual aspect of the mandibular angle contains the mandibular foramen and its associated sulcus, coursing distally from the foramen (hereafter referred to as the *sulcus of the mandibular artery*). A notable characteristic of this area is the large masseteric fossa, which features significantly thinner bone than the surrounding coronoid process, the condylar process, the ventral cortex, and the mandibular body. The masseteric fossa also has a dynamic contour. These unique features make ORIF in this region more challenging than in the mandibular body. Thus, mandibular angle fractures may benefit from additional study and consideration, as has been proposed in human medicine.

Historically, feline caudal mandibular/angle fractures have been managed with non-rigid techniques (e.g., muzzles and modified labial button technique) and semi-rigid techniques (e.g., maxillomandibular fixation with dental composite). However, complications associated with these repair techniques have also been reported (14). For example, techniques that rely on the patient’s own occlusion to stabilize the fracture (e.g., MMF and BEARD) carry inherent risks, such as aspiration pneumonia, due to the inability to open the mouth during vomiting (8, 15). The challenges encountered while managing feline caudal mandibular/angle fractures are mostly due to the small size, irregular contours, and thin nature of the bone of the masseteric fossa, and the sparsity of reliably thick cortical bone for screw anchorage (14). Though challenging, many benefits of ORIF encourage veterinarians to evaluate feasible applications of ORIF in an anatomically challenging region. Recently, a study demonstrated the feasibility of locking and non-locking L-miniplate fixation of simulated caudal mandibular fracture in cats (16).

Fracture patterns may be studied quantitatively or qualitatively. Fracture maps are a qualitative analysis method that has been used in human scapular fractures and feline mandibular fractures to identify and better understand common fracture patterns, in order to facilitate proper ORIF strategies (14, 17). After fracture lines are placed onto a normal template, one can visualize groups of fracture lines distributed on the template. By studying these fracture lines, hardware can be designed for fracture fixation. The aims of this study were to: (1) assess associations between feline angle fracture patterns and demographic characteristics and fracture etiologies and (2) visually analyze feline angle fracture patterns using fracture maps. The hypotheses that we propose are as follows: (1) fracture patterns are associated with different demographic characteristics and fracture etiologies, and (2) fracture maps can elucidate qualitative data that may serve as the basis for additional fracture pattern understanding and for future hardware selection and/or design.

## Materials and methods

### Case identification

Electronic medical records of the University of Wisconsin—Madison Veterinary Medical Center were searched using keywords (feline, cat, mandible, mandibular fracture, head/skull computed tomography (CT) scan, and computed tomography) to identify cats that received a head CT scan between 2008 and 2022.

**Figure 1:**
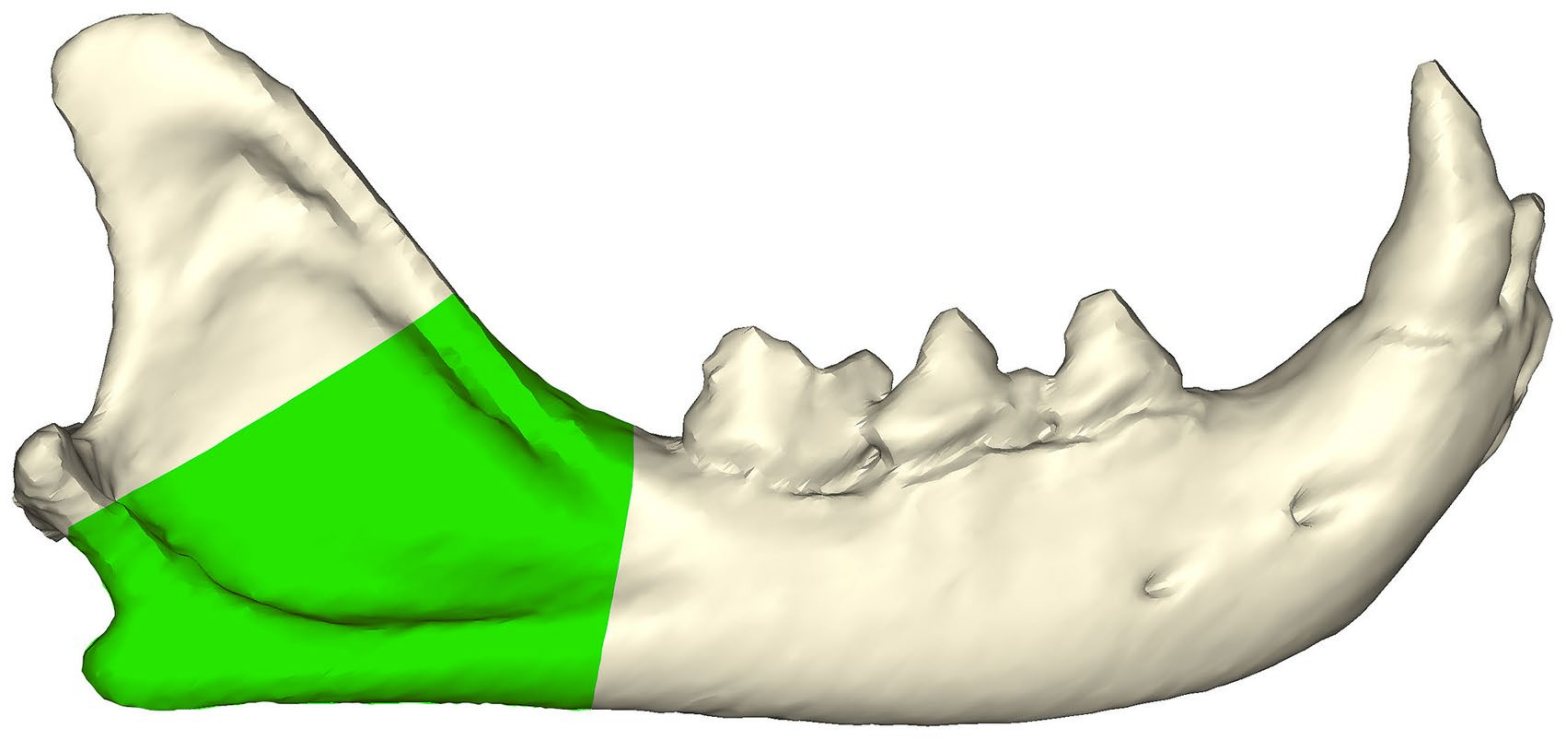
Feline mandibular angle (green zone—viewed from the buccal aspect) was defined as the region between the distal aspect of the mandibular first molar tooth and ventral to the coronoid process.

Cats were included in the study if a diagnosis of mandibular angle fracture was given. The diagnosis of angle fracture was based on the head CT images. Feline mandibular angle fractures were defined for the purpose of this study as fractures in the region between the distal aspect of the mandibular first molar tooth and ventral to the coronoid process (Figure 1). Further screening of the DICOM images to ensure the fractures were in the area of interest was subsequently performed. The exclusion criteria included cats with neoplasms and fractures that were not associated with the mandibular angle. Since this study was retrospective and all patient-identifying information was removed, IACUC oversight was not required.

### Patient demographics, etiology, and fracture characteristics

Medical records, CT images, and radiology reports were reviewed for patients’ signalment (age, sex, and weight), fracture etiology, and fracture characteristics. Fracture characteristics included the following: (1) the side of the fracture, (2) simple vs. comminuted fracture, (3) dorsal fracture location, (4) ventral fracture location, (5) the presence or absence of concurrent mandibular symphyseal separation/parasymphyseal fracture, (6) fracture involvement of the mandibular foramen, (7) number of fracture fragments, and (8) fracture displacement score (18). The displacement score is based on the degree of fracture fragment overlap. Displacement scores of 1, 2, and 3 correspond to no displacement of the fracture, minimal fracture displacement with more than 50% overlap remaining between fragments, and severe fracture displacement with less than 50% overlap remaining between fragments, respectively. The *origin* and the *termination* of each fracture were utilized to describe the dorsal fracture location and the ventral fracture location, respectively. The termination of each fracture was further classified as (1) at, (2) dorsal to, and (3) rostral to the angular process.

### Statistical analysis

A statistical evaluation to look for associations between dependent and independent variables was performed. The dependent variables include fracture type (simple vs. comminuted fracture), dorsal fracture location/fracture origin (ramus vs. mandibular body), ventral fracture location/fracture termination (angular process, dorsal to the angular process, rostral to the angular process, or both dorsal and rostral to the angular process), mandibular foramen involvement (yes/no), and displacement score (<3 vs. equal to 3). The independent variables include age (<1 year and ≥ 1 year), sex, and etiology (animal altercation vs. unknown etiology and other etiology). One year was chosen as the cutoff for age based on the general consensus that cats reach skeletal maturity at approximately 1 year of age. Fisher’s exact tests were used for all analyses. Mixed-effects logistic regression, which accounts for cats with multiple fractures, was also examined and determined to have no change in the statistical results compared to the simpler Fisher’s exact test results. Analyses were conducted using R statistical software (version 4.1). A 5% significance level was used to judge statistical significance.

### Fracture map

CT DICOM images were imported into a three-dimensional modeling software (Mimics Innovation Suite, Materialise, Leuven, Belgium) in order to create a fracture map. Three-dimensional mandibular computer models of each case were created via the segmentation method (19). A mirrored right-sided mandibular computer model was created from each left-sided mandible fracture. An intact right mandibular computer model (case #5) was chosen arbitrarily from the cohort to serve as a ‘normal’ template for fracture mapping. The spline tool in Mimics was used to manually trace three-dimensional fracture lines onto the template model. The individual fracture splines were exported separately as STL files and then select files were imported back into Mimics as solid three-dimensional objects to create fracture maps. Fracture maps were created by consensus for each case by a resident in dentistry and oral surgery (TT) and a board-certified veterinary dentist (JWS). Eight fracture maps were created based on variables of interest (age, etiology, fracture location, and pattern). These fracture maps were then reviewed to assess any visual patterns.

## Results

Two hundred and nineteen cases met the initial search criteria. One hundred and sixty-seven cats were excluded from the database due to mandibular neoplasms. Thirty-three cats were then excluded due to fractures outside the area of interest (mandibular angle) leaving a total of 19 cats with 22 mandibular angle fractures included in the study (Table 1).

**Table 1:** Demographic and fracture characterization data for all cases and fractures (FS—female spayed; MC—male castrated; F—female; M1—molar tooth).

Case number	Age (mo)	Sex	Weight (kg)	Etiology	Affected side	Simple (S) vs. comminuted (C)	Fragments	Displacement score	Dorsal/rostral fracture location		Ventral/caudal fracture location					Ventral fracture location [ventral (V), dorsal (D), angular process (AP), both (B)]	Symphyseal separation	Through foramen
									Immediately distal to M1	Ramus	Rostral to angular process (AP)			At AP	Dorsal to AP (Ramus)			
											At M1 level	Distal to M1	Rostral to M1					
1	12	FS	2.43	Unknown	L	S	2	3		+		+				V	+	+
2	168	FS	3.2	Bit by dog	R	S	2	2		+					+	D		
2	168	FS	3.2	Bit by dog	L	S	2	3		+				+		AP		
3	84	FS	4	Bit by dog	L	C	3	3		+		+				V		+
4	12	MC	3.6	Unknown	L	S	2	3	+			+				V		
5	7	MC	4	Bit by dog	R	S	2	3		+		+				V	+	+
6	60	MC	6	Unknown	R	C	4	3		+		+			+	B	+	+
7	132	MC	6	Bit by dog	R	C	4	3		+		+				V	+	+
8	9	MC	3.6	Hit by a fallen chair	R	C	4	2		+		+				V		+
9	2	F	1.44	Unknown	R	S	2	1		+					+	D	+	
9	2	F	1.44	Unknown	L	C	3	3		+				+		AP	+	+
10	36	MC	5.2	Unknown	R	C	5	3		+		+			+	B	+	+
11	156	FS	5.4	Bit by dog	L	S	2	3		+					+	D		
12	96	MC	6.3	Bit by dog	R	C	3	3		+				+		AP		
13	180	FS	4.2	Jaw caught on a collar	L	S	2	3		+				+		AP	+	+
14	108	FS	3.49	Bit by dog	R	C	4	3	+		+				+	B	+	
15	6	F	3	Bit by dog	L	S	2	3		+		+				V		+
16	4	MC	3.5	Unknown	R	C	4	3		+					+	D		
16	4	MC	3.5	Unknown	L	S	2	3		+		+				V		+
17	4	MC	3.48	Bit by dog	R	C	4	3		+		+				V		+
18	108	MC	4.2	Bit by dog	R	S	2	3		+		+				V		+
19	48	MC	5.85	Bit by dog	L	S	2	3		+				+		AP	+	+

Among these 19 cats, 11 cats (12 fractures) were neutered male (11/19, 57.89%), 6 cats were spayed female (6/19, 31.58%), and 2 cats were intact female (2/19, 10.53%).

Six cats (6/19, 31.58%), accounting for eight fractures (8/22, 36.36%), were under 1 year old, and 13 cats (13/19, 68.42%), accounting for 14 fractures (14/22, 63.63%), were over 1 year old.

Eleven cats (11/19, 57.89%), accounting for 12 fractures (12/22, 54.55%), sustained fractures from animal altercations (i.e., dog bites). Two cats (2/19, 10.52%), accounting for 2 fractures (2/22, 9.09%), sustained fractures due to known low-energy trauma (e.g., jaw caught on leg splint and being hit by a falling chair). The etiology was unknown in six cats (6/19, 31.57%), accounting for eight fractures (8/22, 36.36%).

Sixteen cats (16/19, 84.21%) sustained a unilateral mandibular fracture, and three cats (3/19, 15.78%) sustained bilateral mandibular fractures. Twelve fractures (12/22, 54.54%) were on the right side, and 10 fractures (10/22, 45.45%) were on the left side.

Twelve fractures (12/22, 54.54%) were simple fractures, and ten fractures (10/22, 45.45%) were comminuted fractures. Of the comminuted fractures, three fragments were identified in three fractures (3/10, 30.00%), four fragments in six fractures (6/10, 60.00%), and five fragments in one fracture (1/10, 10.00%).

Nineteen fractures (19/22, 86.36%) had a displacement score of 3 (severe displacement with less than 50% overlap remaining between fragments), while one fracture (1/22, 4.55%) had a score of 1 (no displacement), and two fractures (2/22, 9.09%) had a displacement score of 2 (minimal displacement with more than 50% of the fracture overlap remaining between fragments).

Regarding the fracture ‘origin’ at the dorsal border of the mandible, 2 out of 22 fractures (2/22, 9.09%) were immediately distal to the mandibular first molar tooth, and 20 fractures (20/22, 90.9%) were at the junction of the mandibular body and the ramus. Regarding the ‘termination’ point, 10 fractures (10/22, 45.45%) were rostral to the angular process, five fractures (5/22, 22.72%) were at the angular process, four fractures (4/22, 18.18%) were dorsal to the angular process, and three fractures (3/22, 13.63%) had fracture lines both rostral and dorsal to the angular process.

Twelve fractures (12/22, 50.54%) had concurrent symphyseal separation/parasymphyseal fracture. Fourteen fractures (14/22, 63.63%) had the fracture line propagating through the mandibular foramen.

There was no significant association between any of the dependent variables and any of the independent variables (Tables 2–6).

**Table 2:** Associations regarding age.

Variable	<1 year (*n* = 8)	>1 year (*n* = 14)	*p*-value
Fracture type—simple	4 (50.0%)	8 (57.1%)	1.000
Dorsal fracture location—ramus	8 (100.0%)	12 (85.7%)	0.515
Ventral fracture location			0.436
AP	1 (12.5%)	4 (28.6%)	
Dorsal	2 (25.0%)	2 (14.3%)	
Ventral	5 (62.5%)	5 (35.7%)	
Multiple	0 (0.0%)	3 (21.4%)	
Foramen involved	6 (75.0%)	8 (57.1%)	0.649
Three or more fragments	3 (37.5%)	4 (28.6%)	1.000
Displacement score = 3	6 (75.0%)	13 (92.9%)	0.527

**Table 3:** Associations regarding sex.

Variable	Female (*n* = 10)	Male (*n* = 12)	*p*-value
Fracture type—simple	7 (70.0%)	5 (41.7%)	0.231
Dorsal fracture location—ramus	9 (90.0%)	11 (91.7%)	1.000
Ventral fracture location			0.458
AP	3 (30.0%)	2 (16.7%)	
Dorsal	3 (30.0%)	1 (8.3%)	
Ventral	3 (30.0%)	7 (58.3%)	
Multiple	1 (10.0%)	2 (16.7%)	
Foramen involved	5 (50.0%)	9 (75.0%)	0.378
Three or more fragments	1 (10.0%)	6 (50.0%)	0.074
Displacement score = 3	8 (80.0%)	11 (91.7%)	0.571

**Table 4:** Associations regarding etiology.

Variable	Altercation (*n* = 12)	Other (*n* = 10)	*p*-value
Fracture type—simple	7 (58.3%)	5 (50.0%)	1.000
Dorsal Fracture Location—ramus	11 (91.7%)	9 (90.0%)	1.000
Ventral fracture location			0.93
AP	3 (25.0%)	2 (20.0%)	
Dorsal	2 (16.7%)	2 (20.0%)	
Ventral	6 (50.0%)	4 (40.0%)	
Multiple	1 (8.3%)	2 (20.0%)	
Foramen involved	7 (58.3%)	7 (70.0%)	0.675
Three or more fragments	3 (25.0%)	4 (40.0%)	0.652
Displacement score = 3	11 (91.7%)	8 (80.0%)	0.571

**Table 5:** Associations regarding dorsal fracture location.

Variable	Body (*n* = 2)	Ramus (*n* = 20)	*p*-value
Fracture type—Simple	1 (50.0%)	11 (55.0%)	1.000
Ventral fracture location			0.416
AP	0 (0.0%)	5 (25.0%)	
Dorsal	0 (0.0%)	4 (20.0%)	
Ventral	1 (50.0%)	9 (45.0%)	
Multiple	1 (50.0%)	2 (10.0%)	
Foramen involved	0 (0.0%)	14 (70.0%)	0.121
Three or more fragments	1 (50.0%)	6 (30.0%)	1.000
Displacement score = 3	2 (100.0%)	17 (85.0%)	1.000

**Table 6:** Associations regarding ventral fracture location.

Variable	Non-AP (*n* = 17)	AP (*n* = 5)	*p*-value
Fracture type—Simple	9 (52.9%)	3 (60.0%)	1.000
Dorsal Fracture Location—Ramus	15 (88.2%)	5 (100.0%)	1.000
Foramen involved	11 (64.7%)	3 (60.0%)	1.000
Three or more fragments	7 (41.2%)	0 (0.0%)	0.135
Displacement score = 3	14 (82.4%)	5 (100.0%)	1.000

### Fracture map assessment

#### All fractures

**Figure 2:**
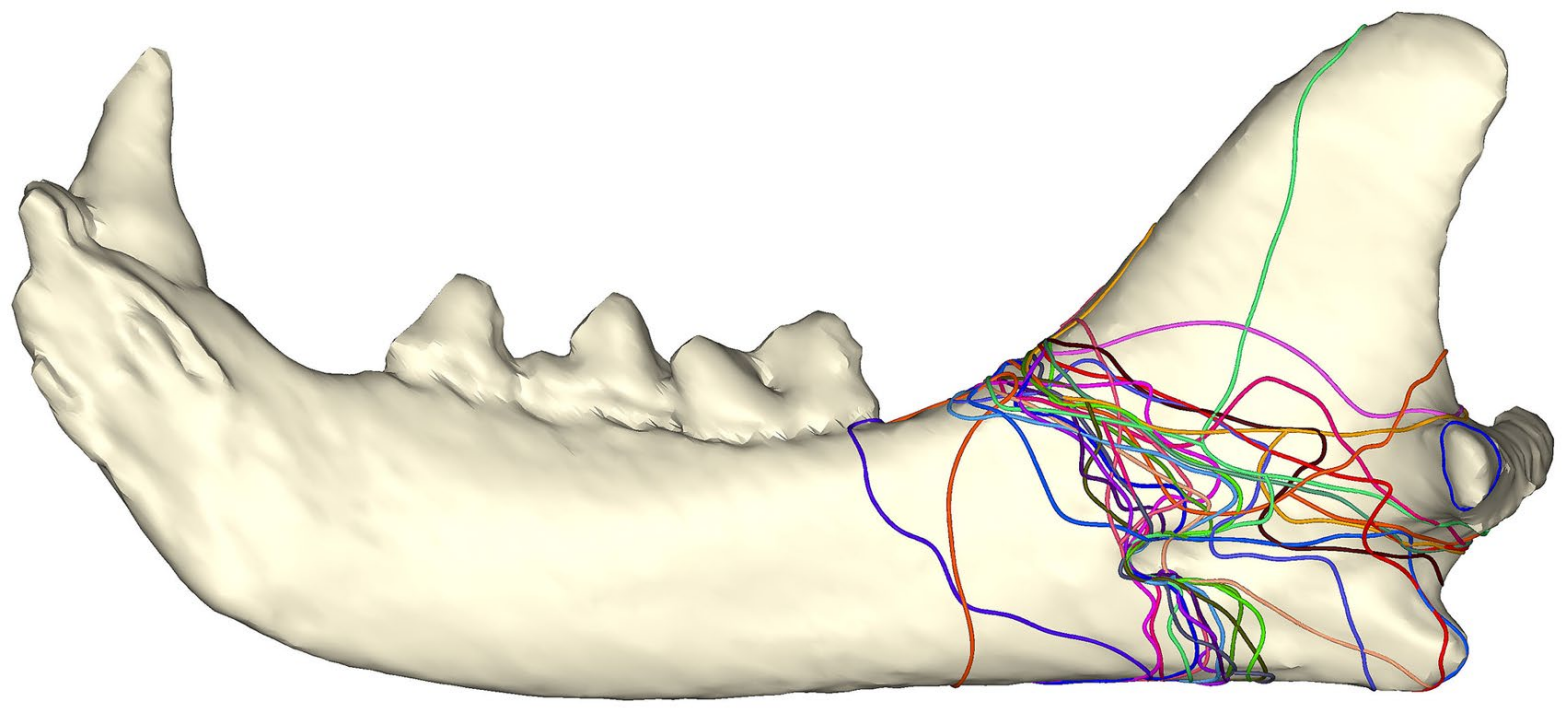
Fracture map depicting all fracture lines viewed from the lingual aspect.

When all the fracture lines were put together, there were two distinct areas that were affected the most (Figure 2). A more distinct pattern is that the fractures originate at the junction of the mandibular body and ramus along the dorsal mandibular border, travel caudoventrally through the mandibular foramen, and terminate at the ventral mandibular border, rostral to the angular process. The second distinct fracture pattern originates at the same location, courses caudoventrally above the mandibular foramen, and terminates dorsal to the angular process, ventral to the condylar neck.

#### Simple vs. comminuted fractures

**Figure 3:**
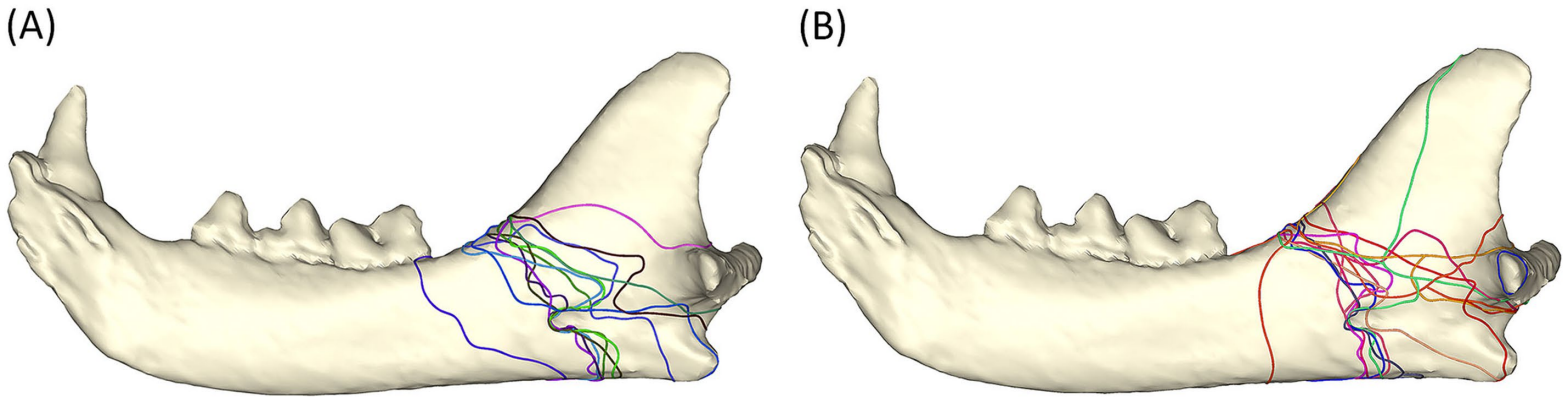
Fracture maps depicting simple fracture lines **(A)** and comminuted fracture lines **(B)** viewed from the lingual aspect. Note that the sulcus of the mandibular artery was less affected by comminuted fractures.

The majority of simple fractures originate from the area where the mandibular body meets the ramus and follow a ‘sigmoid’ fracture pattern through the masseteric fossa, mandibular foramen, and the sulcus of the mandibular artery. Fractures terminated rostral to, at, or dorsal to the angular process (Figure 3A).

The distribution of the comminuted fractures is more chaotic than the simple fractures. The region that is immediately ventral to the condylar neck is more commonly affected than the simple fracture. The fractures still tend to follow a path through the mandibular foramen (although fewer course through the sulcus of the mandibular artery). The affected region is wider than in simple fracture (Figure 3B).

#### Bilateral fractures

**Figure 4:**
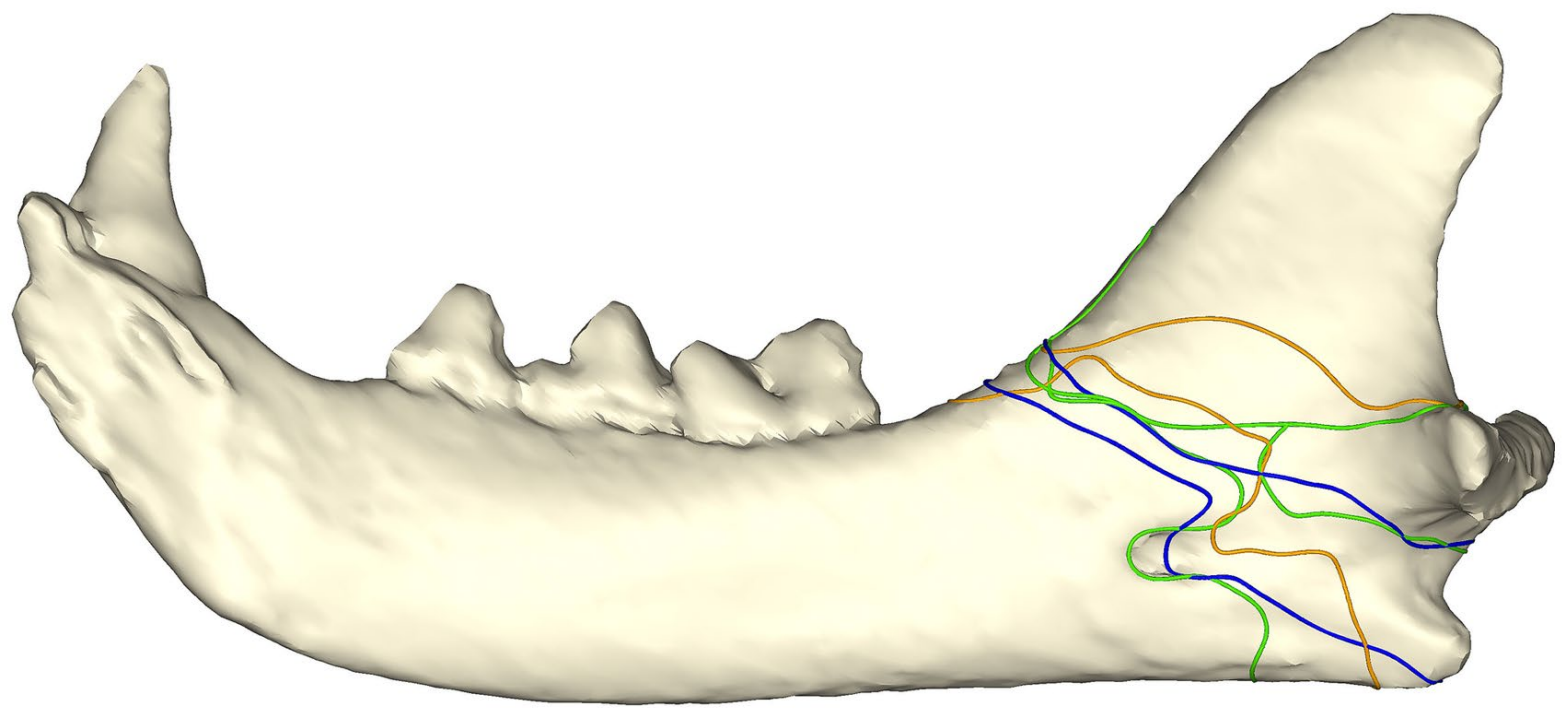
Fracture map with bilateral angle fracture lines in three cats. Both fractures from each cat are depicted on a single mandible with fractures from the same patient depicted with the same color. Note that one fracture from each cat had a sigmoid path involving the mandibular foramen/sulcus of the mandibular artery, whereas the other fracture propagated more horizontally through the ramus.

All the bilateral fractures followed a similar pattern (Figure 4). All the fractures originated at the junction of the mandibular body and the ramus, with the fractures on one mandible possessing a sigmoid fracture pattern through the mandibular foramen or the sulcus of the mandibular artery, and the fractures at the contralateral mandible propagating horizontally and caudally to either dorsal to or ventral to (or both) the condylar neck.

#### Fracture etiology

**Figure 5:**
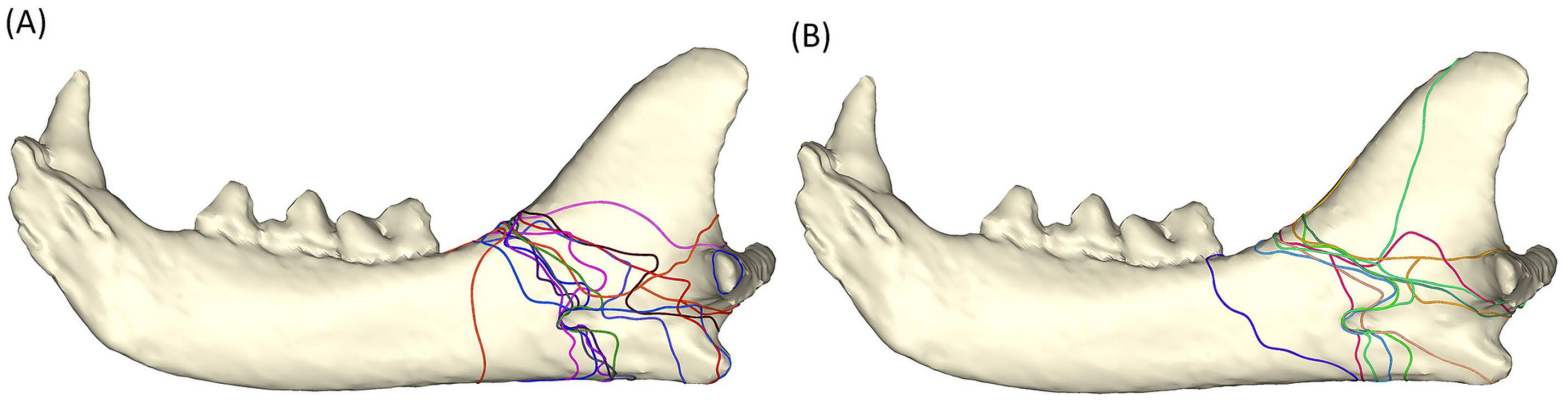
Fracture maps depicting fracture lines in animal altercation patients **(A)** and fracture lines in unknown etiology patients **(B)**.

All fractures caused by animal altercation were caused by dog bites. The majority of the fractures shared a similar fracture pattern, involving the mandibular foramen or the sulcus of the mandibular artery. Among the 12 fractures, only 1 simple and 1 comminuted fracture line terminated dorsal to the condylar neck (Figure 5A).

Fractures of unknown etiology shared a similar pattern to those caused by animal altercations (Figure 5B). However, these fractures appeared to propagate less frequently through the sulcus of the mandibular artery and more through the mandibular foramen. In the buccal view, fractures from animal altercations seemed to have more fracture lines coursing dorsoventrally to the ventral cortex than those of the unknown etiology. There was one fracture line propagating vertically toward the coronoid process. This fracture line is one of the five fracture lines in a comminuted fracture of a 3-year-old, male castrated, 5.2-kg patient (case # 10).

**Figure 6:**
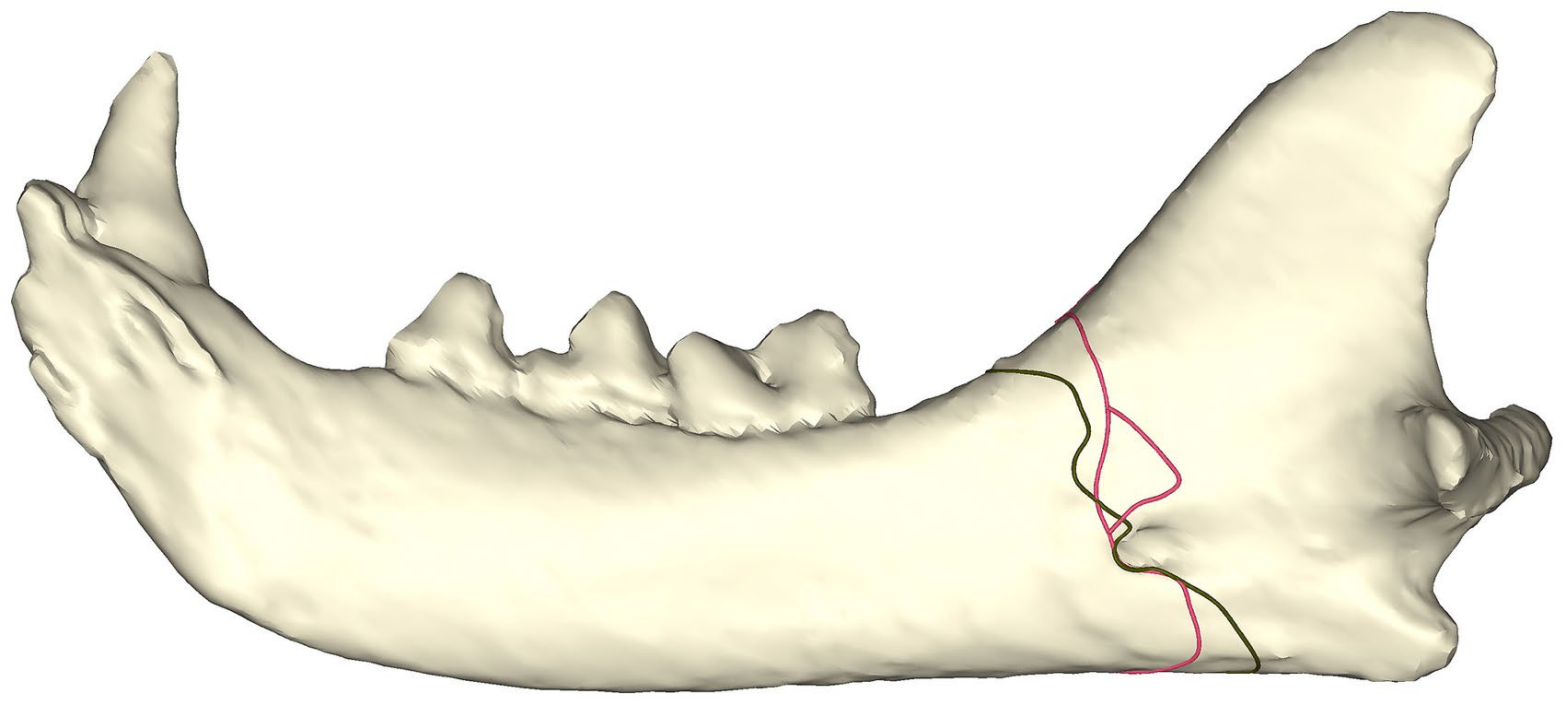
Fracture map depicting fracture lines in low-energy fracture patients.

Both fractures, caused by a low-energy trauma, coursed through the mandibular foramen (Figure 6).

#### Juvenile vs. adult

**Figure 7:**
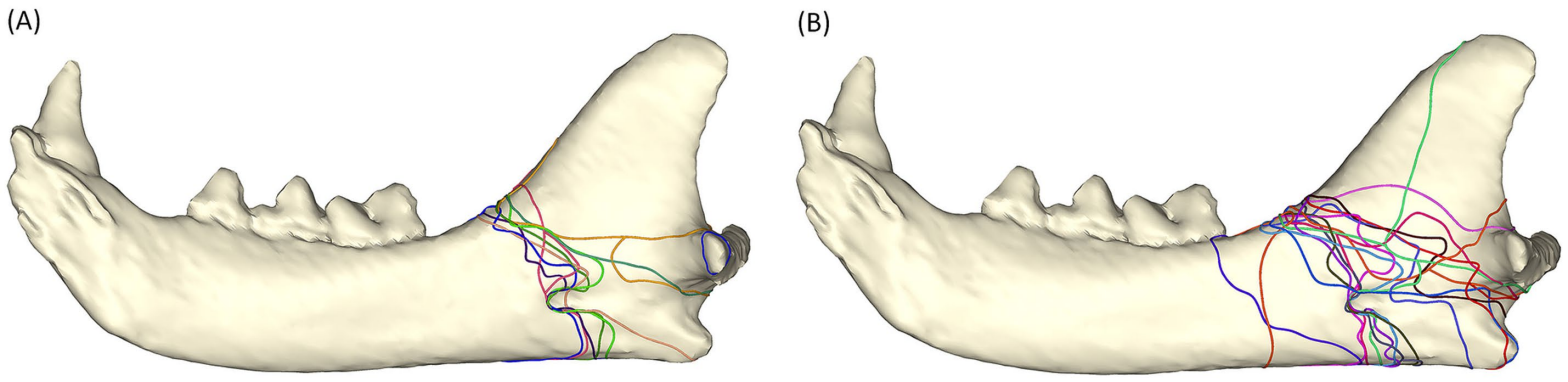
Fracture maps depicting fracture lines in juvenile cats **(A)** and adult cats **(B)**.

The fracture distribution in juvenile cats is similar to that in fractures of unknown etiology, as the fractures typically extend to the mandibular foramen but not the sulcus of the mandibular artery. In contrast, adult cats had more fractures that extended through the sulcus of the mandibular artery (Figure 7).

## Discussion

One of the objectives of the present study was to identify predictive variables related to the locations and patterns of feline mandibular angle fractures. A relatively minor yet important step in the design of this study was defining the mandibular angle, which has historically been poorly defined in cats and dogs. While the mandibular angle has been mentioned in anatomical texts, a clear definition has not been provided (20). In one study of 45 cats presented for head trauma, the term *angle of mandible* was shown in one of the figures (21). However, this term was not further defined, and no information related to this term was provided. Rather than analyzing the *angle of the mandible*, the angular process, condylar neck, and ramus were analyzed as distinct functional anatomical regions (21). The craniomaxillofacial fracture locations, morphologies, and etiologies in dogs have been well described (18). In that study, the canine mandibular angle was referred to as mid-ramus, and the fractures that affected the mandibular angle were associated with the molar part of the mandible (18). Interestingly, the canine mandibular angle was not one of the areas of interest that was used for evaluating concurrent fractures. Another anatomic structure defined in this study was the sulcus of the mandibular artery. Given the potential significance of this structure in feline mandibular angle fractures, it seems prudent that it should have a name. Unfortunately, we have not been able to find a specific name for it in any anatomical reference. By definition, a sulcus is a shallow depression, fissure, or groove. A similar anatomical structure, the palatine sulcus, accommodates the associated neurovascular bundle (22). While the term ‘mandibular sulcus’ was considered, it is already used in human anatomy to describe a different anatomical feature, which could lead to confusion. Thus, we propose the term *sulcus of the mandibular artery*.

No association between dependent and independent variables was found in the present study. In other words, independent variables, such as sex, age, and fracture etiology, in this study could not be used to predict the fracture location or pattern. Possible reasons for the lack of significant associations could include: (1) the small sample size, (2) the high number of unknown fracture etiologies, or (3) the possibility that mandibular geometry may be influencing fracture location and pattern more than the etiology. The fracture maps, however, proved useful in providing qualitative information regarding pattern visualization. We believe this pattern analysis provides insight into the biomechanics of the feline mandibular angle and suggests that mandibular geometry influences the mandibular angle fracture patterns in cats.

The majority (19 out of 22 fractures) of the fractures in this study had a displacement score of 3. This suggests that feline angle fractures would benefit from anatomical reduction and rigid fixation to achieve proper bone healing and prevent malunion and potential temporomandibular extra-articular ankylosis (23). With higher energy, fractures tend to be more comminuted with smaller fracture fragments, which creates a more challenging anatomical reduction (24). Almost half of the feline angle fractures in the present study were comminuted. Seventy percent of the comminuted fractures had greater than three fragments. The use of a single plate for rigid fixation is limited in these highly fragmented cases. Given this finding and the small size of the feline mandible, a more versatile implant is desirable.

The science of material fracture (fracture mechanics) is very complex and highly dependent on the nature of the material. However, at a basic level, material failure (fracture) tends to originate in areas of high stress, propagate through relatively weak (high-stress) structures, and terminate at the boundary of the material (i.e., complete fracture). Due to the retrospective nature of the present study, it is impossible to determine the origin of each fracture. However, based on previously published biomechanical studies, the junction between the mandibular body and the ramus is understood to be a region of stress concentration (25). It is also intuitive that a mandible subjected to a force from dorsal to ventral at the rostral or mid-mandibular body will experience stress concentration in this region due to a lever arm effect (12). In this scenario, mandibular fractures would likely originate at the dorsal aspect of the transition between the body and the ramus then propagate through the thin masseteric fossa, terminating either (1) at the ventral cortex rostral to the angular process, (2) at the angular process, or (3) dorsal to the angular process.

The fracture mechanics of the cat mandible are further complicated by the presence of a fibrocartilaginous symphysis, or synchondrosis, between the left and right mandibles. As a result, the mandibles can move independently, with varying degrees of normal physiological laxity at the symphysis (26, 27).

In this study, three cats were found to have bilateral mandibular angle fractures, with one cat also having a concurrent symphyseal separation. Although there were differences in the presence of symphyseal separation and the causes of trauma—one from an animal altercation and two with unknown causes—the fracture patterns in the bilateral mandibles were very similar. One side showed a sigmoid fracture pattern through the masseteric fossa, while the other side had a horizontal fracture ending near the condylar neck. This may indicate that differences in force magnitude and direction influenced the fracture patterns, or that one side may have fractured before the other. The study identified one case of animal altercation, but the causes of the other two cases remain unknown, making it difficult to draw clear conclusions about the specific nature or direction of the impacts leading to these fractures. It is also possible that force was applied directly to the angle region, either on one side or both. However, this would oversimplify the biomechanics involved, especially since the study was retrospective and did not include direct observations of the trauma’s forces and direction. The complex nature of force direction and mechanism makes it difficult to reach definite conclusions, highlighting the need for future research to better understand the relationship between force application and fracture patterns.

Visually, the fracture map of fractures caused by animal altercation and the fracture map of simple fractures are similar. Whereas fracture patterns of unknown etiology and of comminuted fractures are similar. This may indicate that cases that experienced trauma of unknown etiology may be of a higher energy than animal altercation. However, to better understand the fracture energy and subsequent fracture pattern, a larger number of cases with known etiologies, other than animal altercation, would have been helpful.

One year of age was selected as the cutoff between adult and juvenile patients, as it is generally agreed that cats reach skeletal maturity around this age (28). Fractures in juvenile patients tended to involve the mandibular foramen, compared to adult fractures. This may imply that this distribution represents a vector of the weakest/thinnest bone within the angle region of juvenile cats, and as they mature, this area becomes stronger and thicker due to bone growth (i.e., bone deposition and remodeling).

Interestingly, when the fracture maps were evaluated, differences in the fracture patterns were more easily appreciated from the lingual view. This may suggest that the anatomical structure of the mandibular foramen and the sulcus of the mandibular artery are important anatomical features that influence fracture mechanics within the mandibular angle.

Efforts to classify and analyze mandibular fractures in human and veterinary literature often fail to characterize fracture patterns. Fracture visualization may provide a more intuitive way to characterize these patterns and can assist in the design of appropriate implants (17). Two-dimensional fracture maps can be generated, but they provide limited information. By using a CT image to create a three-dimensional fracture map, the visualization of the fracture presentation is much improved. The present study demonstrates that the mandibular angle anatomy at the lingual aspect may be more critical to fracture patterns of the feline mandibular angle than previously appreciated. In addition, one of the more interesting findings is the ‘sigmoid’ fracture pattern within the masseteric fossa. This fracture pattern could not have been identified based only on textual fracture descriptions.

Biomechanically, the ideal area for implant placement is on the tension side (i.e., the dorsal mandibular border where the mandibular body and ramus meet) of the bone fracture to achieve stabilization and compression. However, the bone quantity (i.e., bone thickness), the unique contour of this region, and the presence of teeth make implant placement challenging. Using a single bone plate on the ventral cortex of the mandible for mandibular body fracture stabilization is biomechanically acceptable in dogs (29). In cats, L-miniplate fixation was studied *ex vivo* (16). In this study, caudal mandibular fractures were simulated with osteotomies rostral to the angular process and were stabilized with either locking reconstruction or non-locking reconstruction plates. Both configurations showed clinically acceptable fracture stability. However, when the fracture terminates dorsal to the angular process, the surgical approach becomes more challenging, and the bone to be used for implant placement becomes less ideal. While one bone plate has been specifically designed to tackle this problem (14), the extensive surgical approach into the masseteric fossa for implant placement may not be necessary. A cuttable implant (e.g., metal mesh) may be an alternative option in terms of customization for each patient. Future biomechanical studies are warranted to assess the feasibility of this type of implant.

One limitation of the present study is the small sample size and large variance, which may have prevented the finding of statistical significance. In addition, the cohort in the present study was from a referral center and may not be representative of the whole affected population. The proportion of etiologies could vary across different geographic regions, and these varying etiologies may further influence fracture patterns due to differences in the energy and direction of the force. The concurrent injury to the inferior alveolar neurovascular bundle or complications associated with the injury (e.g., devitalization of teeth rostral to the fracture and paresthesia) were not evaluated in this study. Another limitation of the present study is that the fracture mechanisms were unknown in many cases. Given the retrospective nature of this study, a description from the witness was unavailable. Furthermore, even when the injury was witnessed (e.g., animal altercation), the actual mechanism (force magnitude and direction) was unknown.

In conclusion, no association was found between feline angle fracture patterns and demographic characteristics or etiologies. Fracture mapping proved very insightful in understanding fracture patterns, particularly when viewed from the lingual aspect of the mandibular angle. This study suggests that geographic/morphologic features of the feline angle may play an important role in influencing fracture patterns. Future biomechanical studies of the feline angle should prove fruitful.

## Data Availability

The raw data supporting the conclusions of this article will be made available by the authors, without undue reservation.
